# Comparison of Different Cells of *Haematococcus pluvialis* Reveals an Extensive Acclimation Mechanism during its Aging Process: From a Perspective of Photosynthesis

**DOI:** 10.1371/journal.pone.0067028

**Published:** 2013-07-26

**Authors:** Wenhui Gu, Xiujun Xie, Shan Gao, Wei Zhou, Guanghua Pan, Guangce Wang

**Affiliations:** 1 Key Laboratory of Experimental Marine Biology, Institute of Oceanology, Chinese Academy of Sciences, Qingdao, China; 2 University of Chinese Academy of Sciences, Beijing, China; 3 College of Marine Science and Engineering, Tianjin University of Science and Technology, Tianjin, China; 4 Marine Fisheries Institute of Jiangsu, Nantong, China; IISER-TVM, India

## Abstract

Both biomass dominated green vegetative cells (GV) and astaxanthin-dominated orange resting cells (OR) affect the final astaxanthin yield in industry. Examination of *Haematococcus pluvialis* revealed that the OR cells greatly varied from the GV cells at both cellular and subcellular levels. In particular, the thylakoid membranes in the OR were disassembled and fragmented. Furthermore, the OR conserved most of the photosynthetic pigments, with elevated concentrations of violaxanthin, antheraxanthin, and neoxanthin. Notably, moderate photosynthesis was detected in OR, even though most of the thylakoid membranes were disassembled, when compared with those in the GV. However, the energy distribution pattern between photosystem I and II (PSI and PSII) in the OR favored PSI, which was also confirmed by 77-K fluorescence. As zeaxanthin was not detected in the OR, we attribute the acclimation role to astaxanthin, instead of xanthophyll cycle. Additionally, proteomic-scale comparison analysis of thylakoids of the OR and GV indicated no photosynthetically remarkable variations. However, an extensive acclimation mechanism of *H. pluvialis* was proposed, in which proteins in thylakoid of GV were noted to be involved in biomass accumulation and those in OR were involved in stress response. Conclusions of the comparative analysis might provide some physiological background of OR for astaxanthin production by using *H. pluvialis*.

## Introduction

*Haematococcus pluvialis* is considered to be one of the most prominent candidates for natural astaxanthin production, making it a valuable organism in commercial application [1]. According to the morphological and physiological changes in its life cycle, *H. pluvialis* cells can generally be divided into two types, the biflagellated green vegetative cells (GV) involved in biomass accumulation and resting cells without flagella involved in astaxanthin synthesis [2]. The resting cells can be further classified into green resting cells (GR), orange resting cells (OR), and red cysts (RC) according to cell hue/color and morphological characteristics [3]. OR cells were in process of rapid astaxanthin accumulation [3] and were also in nutrient starvation. A comparative study of the photosystem (PS) of these cells might also provide physiological background for optimization of astaxanthin production using *H. pluvialis* in industry.

Photosynthetic activity measurements of *H. pluvialis* cells indicated that the activity varied greatly among different cell types [3]. The GV exhibited the highest photosynthetic activity in photosystem II (PSII); Comparatively for OR, though with a significant reduction, still accounted for 74% of that of GV [3]. Considering that the cell proliferation and biomass accumulation of OR are reduced [4], the purpose of maintaining such a high photosynthetic activity is unknown. Recent study by Kristoffersen et al by measuring in vivo fluorescence lifetimes of chlorophyll-*a* and NADPH demonstrated that open chlorophyll reaction centers did not change significantly in astaxanthin-accumulating cells; NADPH was necessary for astaxanthin synthesis in red-stage cells and might involve in photoprotection in stressed cells [5]. Additionally, the impairment in thylakoid membrane structure of nonmotile cells [6] and significant thylakoid membranes alterations in red cells [7] were also previously observed. In those respects, a detailed characterization and comparative analysis of the GV and OR photosynthetic system could help in the elucidation of this unique phenomenon. Most previous research of *H. pluvialis* with photosynthesis involved focused on stress-induced cells but thylakoid membranes on proteomic level were not specifically studied [6], [8].

In the present study, we considered the developmental differences between GV and OR cells by comparison of morphological and biochemical changes including thylakoid membrane status, evaluation of carotenoids and protein levels. The results demonstrated that the morphological changes of OR at the cellular and subcellular levels were significant, e.g., massive astaxanthin esters accumulation, accumulation of starch and lipid bodies, thylakoid disassembly et al. Furthermore, compared to GV, the photosynthetic efficiency of PSI and PSII in OR exhibited differently in aspects like the decrease of Y(I) and Y(II) with increased ratio of Y(I) to Y(II). However, proteomic study of the thylakoid membrane presented modest differences between GV and OR, and some regulatory proteins were identified. Observations and comparative analysis of GV and OR at several levels demonstrated that *H. pluvialis* maintained high photosynthetic activity during astaxanthin accumulation. Lastly, the underlying mechanisms and cellular responsive functions in OR have been discussed.

## Materials and Methods

### 1 Strain, Cultivation, and Sample Collection

*H. pluvialis* Flotow was conserved in our lab. The GV were cultured in MCM medium [9] at 22±1°C with a 12-h light/dark cycle. Light provided by the fluorescent lamps was controlled at 100 µmol photons/m^2^/s. Sterilized bubbles of air containing 430 ppm of CO_2_ were continuously aerated into the culture. The GV were harvested at the exponential stage when all the cells were biflagellated. The OR were obtained by leaving exponentially grown GV in the MCM medium without further nutrient supplementation for approximately 10 days, under the above-mentioned incubation conditions. The cell culture was centrifuged at 1000 *g* for 5 min at 4°C. To eliminate light illumination effects, the algae were harvested in the dark during the cultivation. The cell pellets were resuspended in fresh MCM medium, washed twice, and centrifuged at 1000 *g* for 5 min at 4°C.

### 2 Morphological Observation

Morphological changes were monitored under differential interference contrast microscope (Nikon ECLIPSE 90i) throughout the cultivation period. Cell diameter was measured using NIS Elements software (Version BR3.10 build 578) after high resolution images were acquired. For each type of cells, 4 cells were randomly picked for diameter determination. The collected GV and OR were fixed using 2.5% glutaraldehyde. Thin sections were stained with lead citrate and observed under a transmission electron microscope (Hitachi H-7000 TEM, Japan).

### 3 Chlorophyll Fluorescence Measurements

For chlorophyll fluorescence analysis, fresh GV and OR cell cultures were subjected to computer-controlled chlorophyll fluorometer Dual-PAM-100 (Heinz Walz GmbH, Germany). The samples were dark-adapted for 15 min before the measurement. Both the photochemical quantum yield of PSI and effective quantum yield of PSII, known as Y(I) and Y(II), respectively, were calculated based on the data acquired during induction curve measurement. The induction curve was measurement using the dual channel mode (Fluo+P700). Data of induction curve was acquired by running the automatic preprogrammed standard mode in DualPAM software (version V1.11) with actinic light set at 100 µmol photons/m^2^/s. Four independent biological replicates were obtained for each group.

### 4 Isolation and Purification of Thylakoid Membrane

The thylakoid membrane was isolated as described earlier [10], with minor modifications. Briefly, approximately 4 g of fresh cell pellet (fresh weight) was suspended in 40 mL of pre-cooled (4°C) extraction buffer (0.3 M sucrose, 25 mM HEPES-KOH, 1 mM MgCl_2_, and 0.1 mM ε-amino caproic acid; pH 7.5). In order to disrupt the cells, the suspension was passed through pre-cooled (4°C) High-pressure Cells Press (Xinzhi JG-1A, China) at 1000 psi in less than 20 s. Cell breakage was validated via microscopic observation to ensure that over 95% of the cells were successfully broken. The homogenate was subsequently centrifuged at 1000 *g* for 10 min at 4°C to remove cell debris, cell wall materials, and starch granules. The white pellet (mostly starch) was discarded and the supernatant (containing most of the thylakoid membrane and soluble proteins) was then centrifuged at 105,000 *g* for 30 min at 4°C in a swing-out rotor (Beckman Coulter Optima L-100K, SW32 Ti rotor). The green pellet was resuspended in 40 mL of osmotic buffer (25 mM HEPES-KOH, 1 mM MgCl_2_, and 0.1 mM ε-amino caproic acid; pH 7.5) for 10 min on wet ice to release the soluble stromal proteins and thylakoid membrane. Then, the suspension was subjected to discontinuous sucrose gradient (50, 40, 30, and 20% sucrose, w/v) ultracentrifugation at 105,000 *g* for 4 h at 4°C (Beckman Coulter Optima L-100K, SW40 Ti rotor, USA) for thylakoid purification. The green band between 30 and 40% sucrose gradient layers was collected. To remove sucrose from the thylakoid membrane sample, the collected sample was diluted with 10 volumes of stock buffer (5 mM HEPES-KOH, 10 mM EDTA, and 0.1 mM ε-amino caproic acid; pH 7.5) and ultracentrifuged at 105,000 *g* for 1 h at 4°C (Beckman Coulter Optima L-100K, SW32 Ti rotor, USA). The pellet of thylakoid membrane was stored at −80°C for further analysis. In order to preserve the integrity of the thylakoid membrane and prevent it from protein degradation, all the procedures of thylakoid membrane isolation and purification were carried out under dim light at 0°C, except when specifically mentioned.

### 5 Thylakoid Membrane Solubilization and Spatial Fractionation

Membrane solubilization was carried out according to the protocol described previously [11], with minor modifications. The thylakoid membrane pellet was solubilized in 2 mL of stock buffer (5 mM HEPES-KOH, 10 mM EDTA, and 0.1 mM ε-amino caproic acid; pH 7.5) and completely homogenized. Subsequently, 50 µL of the suspension were mixed with 200 µL of acetone for total chlorophyll quantification (Shimadzu UV-1800). The thylakoid membrane suspension was adjusted to a final chlorophyll concentration of 1 mg/mL. Then, 50 µL of 20% n-dodecyl-β-D-maltoside (DM) solution (w/v) was added to 1 mL of thylakoid membrane suspension, making the final chlorophyll concentration to 1 mg/mL and DM to 1% (w/v). Thus, the ratio of DM to chlorophyll was set as 10∶1 (w/w). The mixer was left on wet ice for 10 min for membrane protein solubilization. Fractionation of solubilized thylakoid membrane was performed according to the method described by Takahashi [12], with minor modifications. Continuous sucrose gradient, from 5% (w/v) to 20% (w/v), containing 0.1% (w/v) DM was prepared and pre-cooled on wet ice. Traces of insoluble materials were removed by centrifugation at 10,000 *g* for 15 min at 4°C. The solubilized protein suspension was loaded onto continuous sucrose gradient and then subjected to ultracentrifugation at 105,000 *g* for 20 h at 4°C (Beckman SW40 Ti rotor, USA). At the end of centrifugation, fractionated bands were carefully removed from the tube and frozen at −80°C for further pigment analysis.

### 6 Pigments Analysis by High-performance Liquid Chromatography

The thylakoid membrane sample was mixed with cold (4°C) pigment extraction solution (acetone:acetonitrile = 1∶1, v/v) and incubated on wet ice for 10 min. With regard to samples collected from gradient sucrose, every 50 µL of the sample was mixed with 950 µL of the extraction solution. All the samples were centrifuged at 10,000 *g* for 10 min at 4°C. The extraction procedure was repeated until a completely white pellet was obtained. The supernatant was stored at −80°C for high-performance liquid chromatography (HPLC) analysis. Three replicates of pigment analysis were conducted as described previously [13], with minor modifications, using HPLC (Agilent 1200, USA) equipped with ZORBAX C18 reverse-phase column (Agilent, 250-mm long, 4.6-mm *Φ*, 5 µm, USA). Briefly, the mobile phase consisted of Solutions A (H_2_O), B (methanol), C (acetonitrile), and D (ethyl acetate). The following elution gradient procedure was employed: 5% of Solution A, 30% of Solution B, and 65% of Solution C for the first 5 min; a linear gradient to 15% of Solution B and 85% of Solution C from 5 to 12 min; a linear gradient to 10% of Solution A, 55% of Solution B, and 35% of Solution C from 12 to 14 min; and a linear gradient to 45% of Solution B and 55% of Solution D from 14 to 30 min. The flow rate was set at 0.8 mL/min. Column temperature was controlled at 20°C and detector wavelength was set at 432 nm. For each HPLC run, 10 µL of the supernatant were used. The pigments were identified on the basis of the retention time of the pigment standards and the absorption spectra under the same separation condition.

### 7 Comparative Analysis of Thylakoid Membranes in GV and OR

For protein separation, the 20-h centrifuged gradient was divided into 19 fractions and resolved by tricine-sodium dodecyl sulfate-polyacrylamide gel electrophoresis (Tricine-SDS-PAGE), as described previously [14]. For better protein resolution, 7–14% linear gradient gel (16×20 cm) produced by a gradient mixer (Bio-Rad 475 gradient delivery system, USA) was used. Gel run was performed at a controlled temperature of 6°C by connecting the electrophoretic system to a refrigerated circulator (Thermo Cool Tech 320). The proteins were subsequently visualized by silver staining as described previously [15]. High-resolution gel images were acquired by using an imaging densitometer (Bio-Rad GS 800, USA), and three replicates were comparatively analyzed via Quantity One gel analysis software (version 4.6.9, Bio-Rad). Gauss Model Trace, indicating the relative abundance of protein bands, was calculated based on the instructions given in the software. Ten of the most significantly upregulated bands were excised for protein identification.

The excised protein bands were tryptic-digested, and the digested peptides were subjected to matrix-assisted laser desorption/ionization-time-of-flight/time-of-flight–tandem mass spectrometry (MALDI-TOF/TOF–MS/MS) (ABI 4800 plus, USA) for MS/MS analysis, as described previously [16]. The proteins were identified by searching the NCBI database using GPS Explorer (ABI, version 3.6). The search taxonomic category was set to green plants. A minimum of four matched peptides were required. It should be noted that as the genome of *H. pluvialis* has not been sequenced, protein identification, conserved domain identification, and protein functional analysis rely on the alignment of homologous protein sequence of NCBI database.

### 8 Low-temperature (77 K) Fluorescence

Samples of GV and OR were dark-adapted for 5 min and collected as described earlier. The collected cell pellets were stored at −80°C for further analysis. PS variation of *H. pluvialis* at GV and OR stages was assessed by chlorophyll fluorescence at 77 K (liquid nitrogen). The 77 K fluorescence emission spectra was monitored using Hitachi F4600 fluorometer (HITACHI, Japan), and the samples were frozen and maintained in Dewar vessel filled with liquid nitrogen. The excitation wavelength was set at 436 nm and slit width was set at 5 nm for both excitation and detection. The emission spectra data from 400 to 800 nm were recorded, and the spectra were normalized to area below the curve from 665 to 735 nm.

### 9 Statistical Analysis

Data were given as mean values ± standard deviation. All statistical data analysis was conducted by SAS 9.1 for Windows software (SAS Institute Inc., USA). One-way analysis of variance (ANOVA) was used to determine the significant difference between groups at *P*<0.05.

## Results

### 1 Cellular and Subcellular Morphological Changes

**Figure 1:**
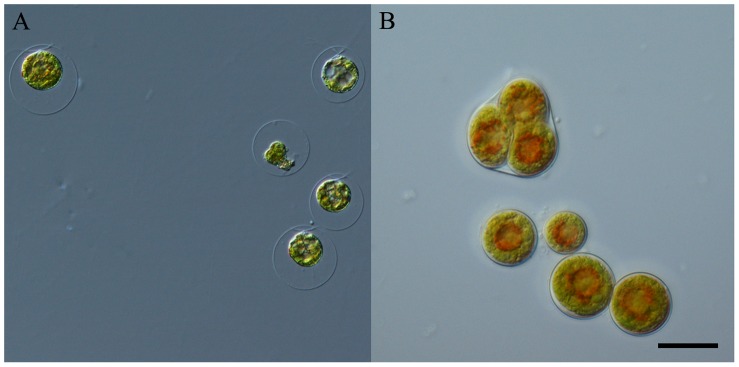
Microscopic observation of GV and OR cells. (A) GV cells were observed to be actively swimming in medium, making GV cells more competitive in cell proliferation and biomass accumulation. (B) OR cells lost the two flagellae and increased in size. The most striking feature of OR was the massive astaxanthin esters accumulated throughout the cells (red colour in OR was mostly from astaxanthin esters). bar = 20 µm.

The GV of *H. pluvialis* were biflagellated (Fig. 1A) and actively swimming in the medium. Whereas, the OR lost the flagella and astaxanthin accumulation spread from the centers to borders throughout the cells (Fig. 1B). In addition, due to the massive accumulation of secondary metabolites such as carotenoids, lipids, starch, etc., the OR were reasonably larger than the GV, with an average increase in cell diameter from approximately 16 to 20 µm.

**Figure 2:**
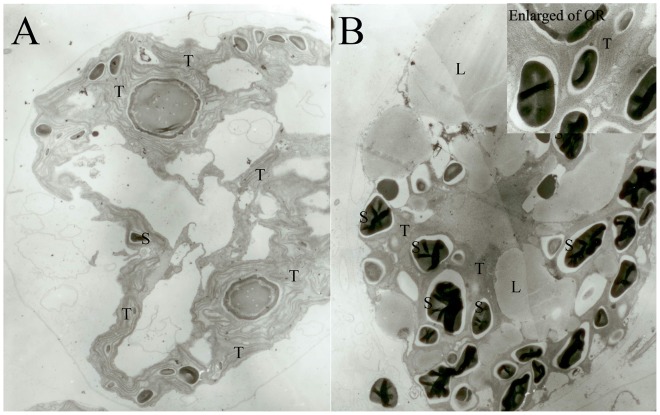
Thin-section electron observations of GV and OR cells. A, GV were rich in continuous thylakoid where photosynthesis took place. A small number of starch granules were observed and almost no lipid body was observed in GV. B, OR showed dramatic decrease in thylakoid volume and significant increase in starch granules and lipid bodies. Thylakoid around starch granules was continuous in OR (the enlarged of OR). S in figure represents starch; T represents thylakoid; L represents lipid bodies.

Thin-section electron observation presented that GV were rich in continuous thylakoid membranes (Fig. 2A). Also, it was noted that several starch granules was observed and no lipid body was observed in GV (Fig. 2A). Comparatively in OR, there was an obvious dramatic decrease in the thylakoid membrane volume, and only a little portion of thylakoid was continuous around the starch granules (Fig. 2B enlarged of OR). Furthermore, as shown in Fig. 2B, an increase in starch granules and large lipid bodies were also observed in OR.

### 2 Chlorophyll Fluorescence Analysis Using PAM

**Figure 3:**
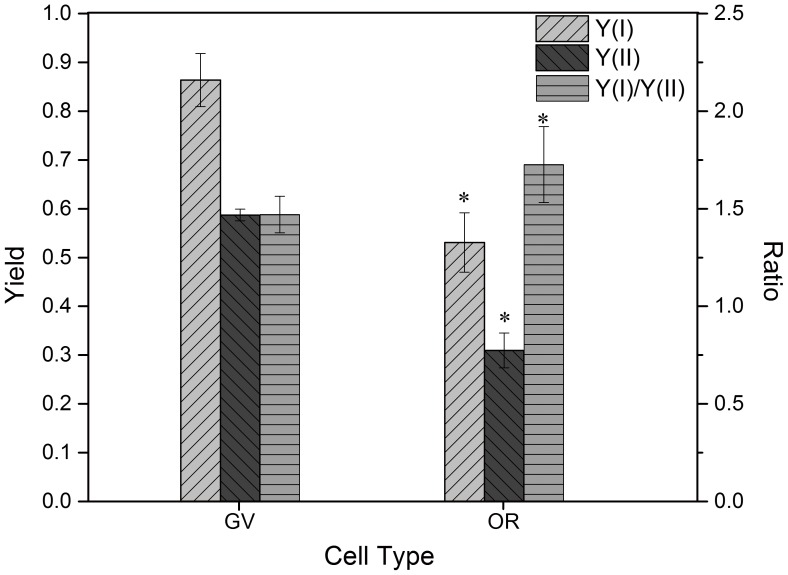
PAM analysis of chlorophyll fluorescence in GV and OR cells. Both Y(I) and Y(II) of GV were comparatively higher than OR. However, for ratio of Y(I) to Y(II), OR (1.72) was higher than GV (1.47). Y(I), Photochemical quantum yield of PS I; Y(II), Effective PS II quantum yield.

Measurement of effective PSII quantum yield, also known as Y(II), showed a value of 0.59 for GV and 0.31 for OR (Fig. 3). With regard to effective PSI quantum yield, known as Y(I), a value of 0.86 was noted for GV, and a comparatively decreased value of 0.53 was observed for OR. However, with respect to the ratio of Y(I) to Y(II), OR exhibited slightly higher value than GV (1.72 and 1.47, respectively; Fig. 3).

### 3 Native Thylakoid Isolation

**Figure 4:**
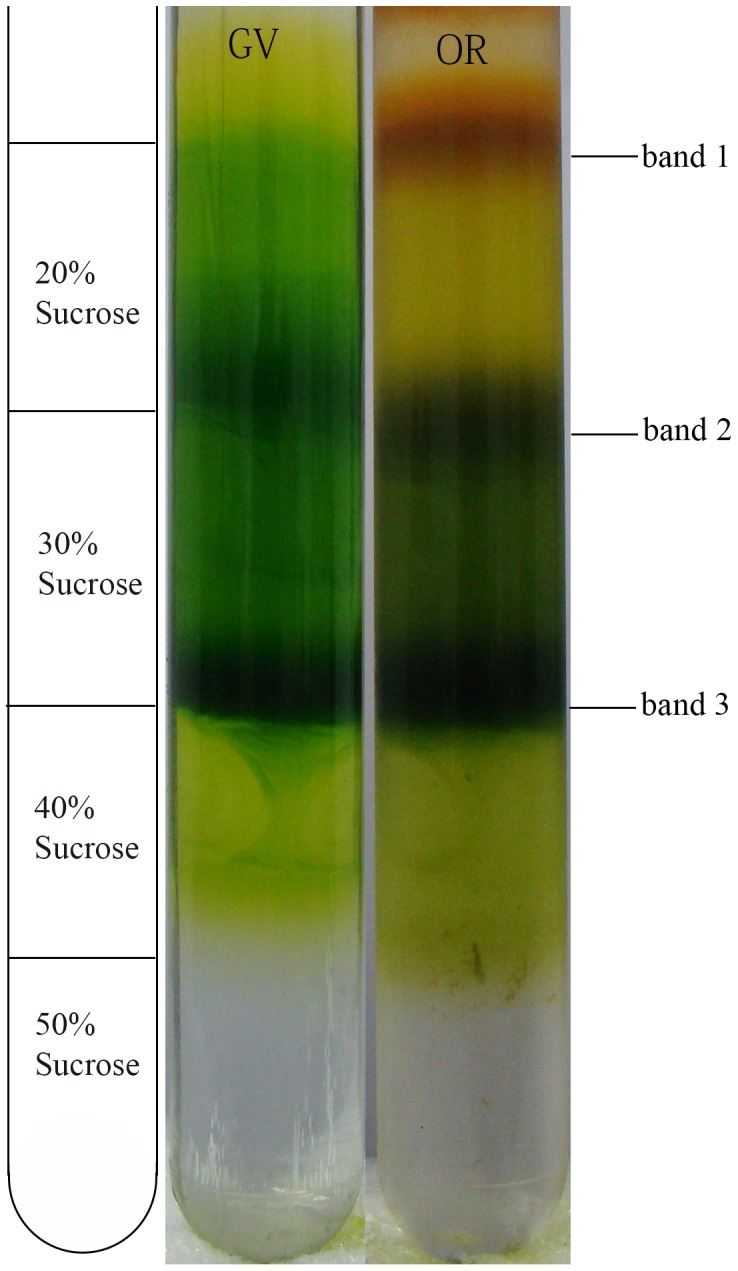
Bands of native thylakoid membrane formed during sucrose gradient ultracentrifugation. Crude thylakoid membrane pellet was subjected to discontinuous sucrose gradient (50, 40, 30, and 20% sucrose, w/v) ultracentrifugation at 105,000 *g* for thylakoid purification. Band 3 was the most abundant membranes stacked between 30 and 40% sucrose layers. Band 2 was fragmented thylakoid and band 1 contained some free pigments released during extraction. Compared to GV, a red band appeared in OR due to the astaxanthin esters released during extraction.

As a mechanical process, high-pressure cell breakage turned out to be sufficient to break over 95% of the OR and nearly 100% of the GV at 1000 psi. Due to the centrifugal property, some free pigments as well as minor lumen and soluble cellular proteins were noted at the top of the centrifuge tube (Fig. 4). At the bottom of the centrifuge tube, some white starch composition released from the thylakoid during ultracentrifugation was observed. Figure 4 shows some fragmented thylakoid membranes stacked between 20 and 30% sucrose layers. For both GV and OR, a visually abundant fraction of thylakoid membrane rich composition was stacked between 30% and 40% sucrose layers. When compared with GV, a red band appeared in OR during thylakoid extraction due to the massive astaxanthin production in OR (Fig. 4).

### 4 Pigments Profiling of Native Thylakoid Membranes

**Figure 5:**
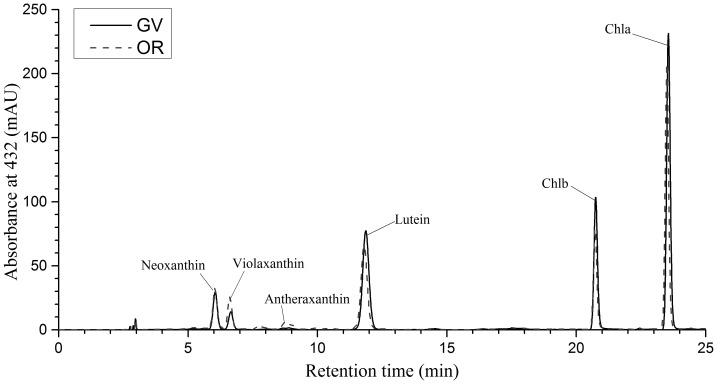
Graph of reverse phase HPLC analysis of pigments in native thylakoid. It was clearly demonstrated that there was similarity of pigments in GV and OR thylakoid. Mean value of three replicates was used. In both GV and OR, chlorophylls were the highest, accounting for 80.38% and 74.29% respectively. Black line represents GV and red lines represents OR.

The composition of the main photosynthetic pigments indicated minor difference between GV and OR thylakoid membranes (Fig. 5). The pigments in GV and OR native thylakoids were both dominated by chlorophylls, as presented in Fig. 5, at a proportion of 80.38% and 74.29%, respectively. However, quantitative analysis on the basis of chlorophylls indicated that the primary carotenoids, including neoxanthin and violaxanthin were statistically different between GV and OR thylakoids; while lutein was not statically different between GV and OR. The level of violaxanthin in OR thylakoid was higher (*P*≤0.05) than that in GV thylakoid (Table 1).

**Table 1:** Quantitative analysis of pigments adhered to native thylakoid membranes.

	GV	OR
N/Chl	0.103±0.011	0.126±0.006
V/Chl	0.049±0.009	0.102±0.009
A/Chl	0.012±0.003	0.050±.010
L/Chl	0.410±0.054	0.402±0.074

N, neoxanthin; V, violaxanthin; A, antheraxanthin; L, lutein.

### 5 Solubilization and Fractionation of Thylakoid Membranes

**Figure 6:**
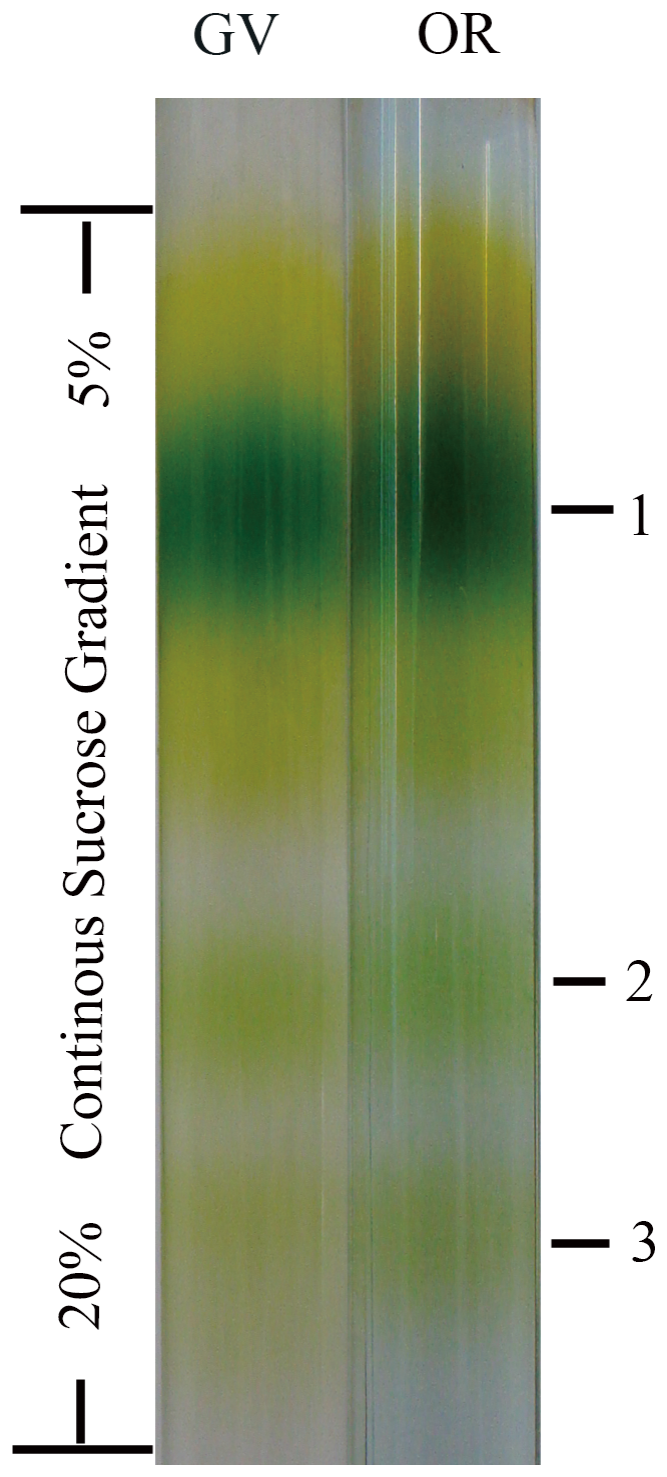
Bands formed by dissociated photosynthesis complexes. Purified thylakoid membranes were solubilized by a mild detergent DM (*n*-dodecyl-*β*-D-maltoside). Subsequently, the continuous sucrose gradient (from 5% to 20%, w/v) ultracentrifugation was used to spatially fractionate the complexes. Three green bands were formed during continuous sucrose gradient ultracentrifugation, namely band 1, 2 and 3 from top to down.

DM, as a neutral mild detergent, dissociated the thylakoid membrane proteins into individual complexes. These dissociated complexes were further fractionated into three separate green bands according to their density by continuous sucrose gradient ultracentrifugation for 20 h (Fig. 6). The three bands were named as Band 1, 2, and 3 from top to bottom, respectively. The results demonstrated that thylakoids from both GV and OR formed three separate bands exhibiting exactly the same pattern in 5–20% sucrose tube.

### 6 Comparative Analysis of Thylakoid Membrane Proteins from GV and OR

**Figure 7:**
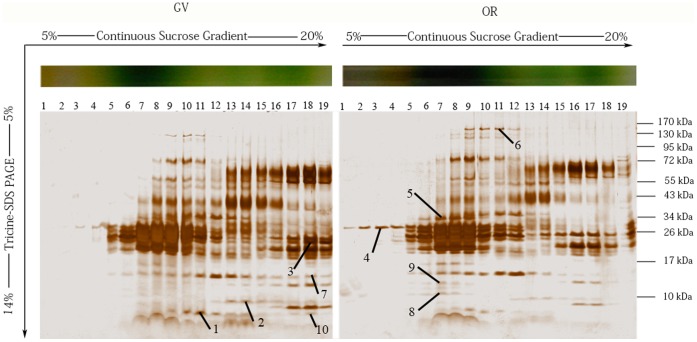
Representative map of protein bands resolved by ultracentrifugation followed by SDS-PAGE. Membrane proteins from GV and OR were fractionated by sucrose ultracentrifugation followed by 7%–14% linear gradient SDS-PAGE. Protein bands were visualized by silver staining. Three replicates for each group were quantitatively analyzed using Quantity One gel analysis software (version 4.6.9, Bio-Rad). 10 most upregulated protein bands were numbered from 1 to 10 as shown in the figure. kDa, kilo-dalton(s).

The silver-stained gel, shown in Fig. 7, presented a map of representative protein bands resolved by ultracentrifugation followed by SDS-PAGE. As presented, the membrane proteins fractionated by sucrose ultracentrifugation were well resolved in SDS-PAGE. The results demonstrate a considerable similarity between the bands obtained from GV and OR (Fig. 7). The upregulated bands (ratio_GV/OR_ ≧3 or ratio_GV/OR_ ≤0.33) are listed in Table 2. Among them, Bands 7 in lane 18 of GV, were significantly upregulated, which were barely detected in the corresponding lane of OR. Consequently, the ratios of Bands 7, was calculated as +∞, while those of the other bands varied from 0.07 to nearly 40 (Table 2).

**Table 2:** Quantitative comparison of the ten most upregulated proteins in GV and OR thylakoid.

Band No. in Fig. 6	Ratio calculated by	Ratio
1	GV/OR	7.09±1.27
2	GV/OR	3.20±0.76
3	GV/OR	39.24±7.06
4	GV/OR	0.17±0.04
5	GV/OR	0.07±0.02
6	GV/OR	0.29±0.02
7	GV/OR	+∞
8	GV/OR	0
9	GV/OR	0
10	GV/OR	10.21±1.82

Ratios were calculated from original data acquired from Quantity One analysis results.

### 7 Protein Identification and Functional Analysis

Ten tryptic-digested samples were successfully identified by MALDI-TOF/TOF MS, and their detailed information is presented in Table 3. Among the 10 proteins, five were from GV and five were from OR. Bands 1, 2, and 3 from GV were demonstrated to be involved in transcription-related process, while Bands 7 and 10 were noted to be involved in secondary metabolisms.

**Table 3:** Protein list identified by MALDI-TOF/TOF MS.

Band No.in Fig. 6	AccessionNo.	Protein name	Samplefrom	Proteinscore	Peptidecount	Protein molecularmass (Da)	ProteinpI
1	gi|40643895	RNA polymerase II second largest subunit	GV	30	5	14972.6	9.3
2	gi|38346261	OSJNBa0074B10.11	GV	44	17	141492.1	9.16
3	gi|7638022	reverse transcriptase	GV	57	9	61949.5	6.03
4	gi|18404536	bacterial hemolysin-related protein	OR	40	9	33183.6	9.23
5	gi|15225404	protein ralf-like 14	OR	40	8	11711	9.42
6	gi|15224353	molybdopterin synthase catalytic subunit	OR	36	6	22202.3	5.76
7	gi|18403524	phospholipase A1-Igamma3	GV	42	10	60283.1	6.18
8	gi|122770	hemoglobin-2	OR	30	5	17834.3	8.93
9	gi|58396949	subitilisin-chymotrypsin inhibitor	OR	36	5	7693	6.23
10	gi|45737905	isopentenyl/dimethylallyl diphosphate isomerase	GV	45	6	10760.7	6.34

Da, Dalton, unit of molecular mass; pI, protein isoelectric point.

With regard to the upregulated proteins obtained from GV, Band 1 was identified as RNA polymerase II second largest subunit, which could catalyze the DNA-dependent polymerization of RNA. Band 2 was found to be homologous to a protein product encoded by a gene from rice chromosome 4, which was observed to contain an RNA/DNA hybrid binding site and a putative NTP binding site. Band 3 was identified as a reverse transcriptase from *Picea glauca*, which was noted to be an RNA-dependent polymerase containing an RNA/DNA hybrid binding site. Bands 7 and 10 from GV were identified as phospholipase A1-gamma 3 and isopentenyl/dimethylallyl diphosphate isomerase (IPP isomerase), respectively. Lipase is an esterase that can hydrolyze long-chain acyl-triglycerides into di- and monoglycerides, glycerol, and free fatty acids at the water/lipid interface.

With regard to the upregulated bands obtained from OR, most of the proteins identified were related to stress response. Band 4 was identified as a bacterial hemolysin related protein. Cross-species homology searches demonstrated that this protein contains the RNA-binding domain found in stress proteins. Band 5 was noted to be a rapid alkalization factor that could elevate the pH of cells when subjected to stress environment. Molybdopterin synthase catalytic subunit identified from Band 6 was observed to belong to MoaE family, which is essential for the diverse group of redox enzymes. Band 8 was identified as hemoglobin-2, with a function of oxygen transportation. Band 9 was observed to be a subtilisin-chymotrypsin inhibitor, and homology searches indicated that this protein is involved in the response to some rice infections.

### 8 Low-temperature Fluorescence

**Figure 8:**
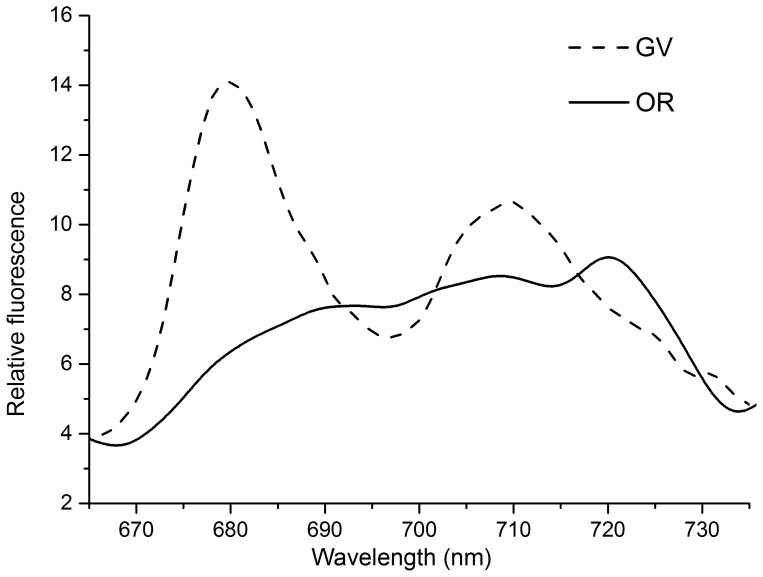
Low temperature (77K) fluorescence emission spectra. The excitation wavelength was set at 436 nm and slit width was set at 5 nm for both excitation and detection. Spectra were normalized to area below the curve from 665 to 735 nm. Two prominent chlorophyll fluorescence emission peaks were observed for GV – a peak at 680 nm from PSII and peak at 710 nm from PSI; the peak at 680 nm was found to be much higher than that at 710 nm. However, OR contained no comparable peaks to GV. Dashed line represents GV and solid line represents OR.

Fluorescence emission spectra at 77 K showed significant differences between GV and OR. As shown in Fig. 8, two prominent chlorophyll fluorescence emission peaks were observed for GV – a peak at 680 nm from PSII and peak at 710 nm from PSI; the peak at 680 nm was found to be much higher than that at 710 nm. On the other hand, with regard to OR, the relative fluorescence emission intensity at 680 nm was lower than that at 710 nm (Fig. 8).

## Discussion

### 1 OR, with Fragmented Thylakoid Membranes, Still Maintained Moderate Photosynthesis

Transformation from GV to OR not only causes loss of flagella, thickening of cell wall, and accumulation of starch and lipids (Figs. 1 and 2), as described previously [8], [17], [18], but also leads to the disassembly of the thylakoid system (Fig. 2B). As the GV exhibit rapid cell proliferation, they are expected to be filled with continuous thylakoids to achieve efficient photosynthesis. Most of the continuous thylakoids observed in GV could barely be detected in OR; however, most of the thylakoid membranes in OR were fragmented and only those around the starch granules were continuous (Fig. 2, the enlarged of OR). Encysted cells such as OR, have been mostly obtained by exposing GV to stress such as strong irradiance [6], [19], thus cell bleaching were mostly occurred ([20]). Nevertheless, in the present study, OR were continuously cultured from GV under the same irradiance, and both cellular and subcellular morphological changes corresponded to the previous observations of stress-induced cells except that few bleached cells in OR was observed. This indicates that depletion of nutrients could also cause degradation of thylakoids in a rather mild manner, unlike strong irradiance. Also, the photosystem balancing scheme of OR thylakoid might not as other species like *Chlamydomonas reinhardtii*, which formed an elusive supercomplex for photosystem balancing between PSI and PSII [21]. With long-term acclimation, OR were able to acclimate and maintain moderate photosynthesis even when most of the thylakoids were disassembled (Fig. 3). However, the photosynthetic characteristics of OR differed from those of GV in several aspects, including the altered Y(II) and Y(I).

### 2 Most of the Photosynthetic Pigments were Conserved in OR with Several Elevated Carotenoids

Most of the photosynthetic pigments, including Chl *a*, Chl *b*, lutein, violaxanthin, antheraxanthin, and neoxanthin were detected in both GV and OR with minor qualitative alterations (Fig. 5). These pigments, adhered to the native thylakoid membranes, are essential, and their relative proportion reflects the energy distribution pattern of photosynthesis. The level of violaxanthin, one of the major carotenoids and a final product of xanthophyll pathway, was significantly increased in OR (Table 1). In addition, the level of antheraxanthin, a major xanthophyll cycle carotenoid, was 4.3 times higher in OR than that observed in GV. However, as zeaxanthin was not detected in either GV or OR, it was concluded that xanthophyll cycle might not function similar to that noted in higher plants [22]. Nevertheless, even without a functional xanthophyll cycle in OR, the resting cells still showed higher tolerance to photo damage [6]. Additionally, lutein in GV and OR maintained relatively constant and it was reported that lutein could substitute zeaxanthin in nonphotochemical quenching during stress [23]. We prefer to attribute this observation largely to the massive level of astaxanthin operating as a light shield in OR. Moreover, astaxanthin could quench the reactive oxygen species (ROS) generated in stress response [6]. As a result, it makes more sense to state that astaxanthin accumulation, instead of xanthophyll cycle, is a more efficient part of acclimation mechanism in OR. Furthermore, as described by Lemoine and Schoefs [24], increased level of violaxanthin also resulted in its participation in astaxanthin synthesis in the putative pathway.

### 3 OR Redistributed Energy between PSI and PSII during Long-term Acclimation

Pulse and amplitude modulation chlorophyll fluorescence indicated that GV and OR greatly varied in PSI and PSII activity. A higher value of Y(II) in GV signifies a much higher efficiency in converting absorbed quanta into chemically fixed energy by PSII. As previously reported, accompanied by thylakoid disassembly, the photosynthetic activity of PSII was observed to decrease during the encystment process [3], [19]. A decrease in cytochrome *f* is considered to be a direct cause of photosynthetic alteration [19]. Accordingly, both Y(I) and Y(II) were noted to decrease in OR, when compared with GV. However, the ratio of Y(I) to Y(II) increased from 1.47 to 1.72, implying a higher activity of PSI in OR, which was also observed in stressed macro-algae [25]. The ratio of Y(I) to Y(II) corresponds with the electron flow ratio of PSI and PSII (known as ratio of ETRI to ETRII). And the simultaneous measurement of both Y(I) and Y(II) provided the energy conversion distribution profile. Previous research [25] found that PSI favored macro-algae showed higher tolerance to stress like desiccation. Thus, it is likely that the balance between PSI and PSII had been altered in OR of *H. pluvialis*; it is possible there is a greater need for ATP production and cyclic photophosphorylation during astaxanthin synthesis.

Marginal changes in chlorophyll fluorescence were observed in most of the cases of cell acclimation, and this technique is considered to be rapid and sensitive for evaluating both long-term acclimation and short-term stress response in the photosystem [26]. At 77 K, fluorescence at 685–695 nm was found to generally originate from PSII antenna pigments, while at 710–735 nm, fluorescence developed from PSI antenna pigments [27]. Thus, 77-K fluorescence intensity is mostly used to assess the relative abundance of the two photosystems. The 77-K fluorescence spectra of GV and OR demonstrated an uneven distribution of the two photosystems: The GV showed relatively higher abundance in light-harvesting complex II (LHCII)–PSII, while OR contained no comparable peaks to GV(Fig. 8). As both GV and OR were dark-adapted at the same level before the measurement, short-term induced (also known as state I/state II transition) fluorescence alterations could be ruled out.

### 4 Some Regulatory Proteins were Observed in OR Thylakoid Membrane, while most Photosynthetic Proteins were Little Altered

Despite the vast morphological and photosynthetic differences between GV and OR, fractionated thylakoids showed a rather similar pattern (Fig. 6), and those of the most abundant proteins were related to photosynthesis. Further detailed comparison through proteomic profiling by SDS-PAGE demonstrated that GV and OR were quite similar to each other. In addition, the differentially expressed proteins that we identified were found to be involved in the physiological regulation processes, instead of photosynthesis. The upregulated proteins identified in GV were observed to be involved in RNA synthesis (Band 1), putative NTP binding (Band 2), reverse transcription (Band 3), and metabolic-related processes (Bands 7 and 10), reflecting a rapid and active cell proliferation process in GV. In addition, these proteins also correspond to the characteristics of abundant accumulation of biomass in the GV, e.g., the upregulated RNA polymerase detected in GV thylakoid was demonstrated to be involved in chloroplast biogenesis and development [28]. Comparatively, the upregulated proteins identified in OR were found to be involved in RNA binding in stress proteins (Band 4), pH elevation during stress response (Band 5), redox regulation (Band 6), oxygen transportation (Band 8), and response to some rice infections (Band 9). All these proteins identified in OR were noted to be closely related to stress response. Especially, proteins involved in redox state regulation, oxygen transportation and pH regulation were detected in OR thylakoid. With nutrients gradually being consumed, OR of *H. pluvialis* encountered stress environments and cell proliferation was reduced. Interestingly, the proteins involved in stress defense have also been detected in soluble proteins obtained from resting cells of *H. pluvialis* [29]. Based on our observation, it seems that some stress-related proteins might also be adhered to the thylakoid membrane to participate in stress defense in order to provide extensive protection to the photosystem.

### Conclusions

In principle, our results demonstrate that there exists a complicated as well as an extensive acclimation mechanism in *H. pluvialis* during its transformation process from biomass-dominated GV to astaxanthin-dominated OR. Morphological changes in OR including thicken of cell wall, accumulation of starch and lipid were found to not only provide a preferential condition for astaxanthin accumulation, but also greatly enhance cell tolerance to stress environment. During astaxanthin accumulation in OR, the Y(I) and Y(II), which indicate photosynthetic activity, accounted for 52% and 62% of GV respectively. However, the stoichiometry between LHC I–PS I and LHC II–PS II was readjusted, in which LHC I–PS I was favored in OR. In this way, the energy absorbed was redistributed between the two photosystems. Consequently, it was possible that OR cells readjusted itself so that the photon-driven process could continuously provide energy for astaxanthin synthesis. Additionally, the regulatory proteins adhered to the thylakoid membranes were also found to involve in stress responses. These above-mentioned regulatory mechanisms constitute an extensive acclimation mechanism in *H. pluvialis* during transformation from GV to OR.
